# Gene Carriers and Transfection Systems Used in the Recombination of Dendritic Cells for Effective Cancer Immunotherapy

**DOI:** 10.1155/2010/565643

**Published:** 2010-12-20

**Authors:** Yu-Zhe Chen, Xing-Lei Yao, Yasuhiko Tabata, Shinsaku Nakagawa, Jian-Qing Gao

**Affiliations:** ^1^Institute of Pharmaceutics, College of Pharmaceutical Sciences, Zhejiang University, Hangzhou, Zhejiang 310058, China; ^2^Department of Biomaterials, Field of Tissue Engineering, Institute for Frontier Medical Sciences, Kyoto University, 53 Kawanara-cho, Shogoin, Sakyo-ku, Kyoto 606-8507, Japan; ^3^Department of Biotechnology and Therapeutics, Graduate School of Pharmaceutical Sciences, Osaka University, 1-6 Yamadaoka, Suita, Osaka 565-0871, Japan

## Abstract

Dendritic cells (DCs) are the most potent antigen-presenting cells. They play a vital role in the initiation of immune response by presenting antigens to T cells and followed by induction of T-cell response. Reported research in animal studies indicated that vaccine immunity could be a promising alternative therapy for cancer patients. However, broad clinical utility has not been achieved yet, owing to the low transfection efficiency of DCs. Therefore, it is essential to improve the transfection efficiency of DC-based vaccination in immunotherapy. In several studies, DCs were genetically engineered by tumor-associated antigens or by immune molecules such as costimulatory molecules, cytokines, and chemokines. Encouraging results have been achieved in cancer treatment using various animal models. This paper describes the recent progress in gene delivery systems including viral vectors and nonviral carriers for DC-based genetically engineered vaccines. The reverse and three-dimensional transfection systems developed in DCs are also discussed.

## 1. Introduction

Cancer is a leading cause of death worldwide. Although progress has been made in cancer therapy with conventional treatment modalities, such as surgery, chemotherapy, and radiotherapy over the last several decades [1], the total number of cancer-related deaths is still increasing. Therefore, there is an urgent requirement to develop novel therapies for the treatment of cancer. With the rapid developments in the fields of immunology and cancer biology, immunotherapy is expected to play a key role in next-generation cancer treatment. The goal of immunotherapy is to promote the patient's own immune system to kill cancer cells instead of using external helpers, that is, surgery or medicine. To induce a specific immune response against cancers, researchers have designed a variety of antitumor vaccines based on the molecular identities of tumor-associated antigens (TAAs). Recent findings from this line of research suggest that immunotherapy strategies are feasible and promising [2–4]. 

DCs are professional and the most potent antigen-presenting cells (APCs) of T-cell special responses, which play an important role in initiating and regulating adaptive immune responses [5]. The major function of DCs in immune system is capturing exogenous and endogenous antigens when infection or cancer occurs, and then presenting the antigens to T cells via major histocompatibility complex (MHC) molecules [6]. Moreover, DCs are also involved in regulating immune tolerance and clonal selection [7, 8]. In 1990, it was firstly reported that injection of DCs with protein antigens *ex vivo* could prime antigen-specific response in animal model [9]. After that, several studies demonstrated that DCs pulsed with TAAs could produce significant therapeutic immunity to tumors with low toxicity. Because DCs could manipulate the immune system by enhancing specific responses to infectious diseases and cancer, DCs networking system became an attractive approach in cancer therapy [10, 11].

The results of early studies in animal models and some preclinical trials indicated that TAA-presenting DC might be a promising treatment for cancer. However, it is difficult to induce long-term tumor-specific immune response in humans. This may be due to the fact that most TAAs are self-antigens, which make cancer cells bypass normal immune protective mechanisms. Therefore, in order to overcome tolerance against self-antigens, it is necessary for an efficient vaccine to induce autoimmune responses [12]. Additionally, the suppressive mechanisms in tumor microenvironment can also inhibit immune response to malignant cells [13]. Hence, designing and developing an efficient and long-term DC vaccine, which could specifically target cancer cells, is urgently needed. Subsequent studies have shown that vaccination using DCs *in vitro* transferred with transgene encoding TAAs or immunomodulatory proteins are more efficient than using cells directly pulsed with protein antigens, tumor peptides, lysates, or RNA [14]. 

This paper focuses on the recent findings in DC vaccinations genetically engineered by recombination biotechnology via different vectors and overviews the development of gene delivery systems for DCs.

## 2. Biological Characteristics of DCs and the Process of DC-Mediated Immune Response

The DCs are generated from CD34^+^ bone marrow stem cells and from DC precursors in the peripheral blood. The concentration of DCs in normal tissue and blood is very low, which makes it difficult to isolate DCs directly from peripheral blood and bone marrow. Currently, the prevalent procedure is to differentiate the monocytes from peripheral blood and bone marrow to DCs with the help of leukapheresis technology and stimulation by cytokines [15].

According to biological properties of DCs, they could be divided into three major groups: plasmacytoid DCs (pDCs), inflammatory DCs (iDCs), and conventional DCs (cDCs) [16, 17]. cDCs are also named myeloid DC (mDCs) owing to their typical form and function [18]. They can be further divided into lymphoid-tissue-resident DCs (splenic, thymic DCs, etc.) and migratory DCs (Langerhans cells, dermal DCs, etc.) [19]. Unlike migratory DCs, which migrate through the lymph, lymphoid-tissue-resident DCs are located mostly at lymphoid tissues to collect and present antigens [20]. Both of the mDCs can be further classified based on the levels of phenotype protein expression and function. For example, CD8^+^ and CD4^+^ mDCs, which were found to preferentially express MHC I and II, respectively, induce different types of T-cell responses [21, 22]. 

On the other hand, on the basis of the different phenotype and surface antigens, DCs could also be divided into immature and mature DCs. The term “immature” refers to DCs with the phenotypic features of low expression of MHC II and molecules such as CD86. In contrast, mature DCs are characterized by high expression of MHC II and T-cell costimulatory molecules such as CD40, CD80, CD83, and CD86 [23–25]. Under pathologic conditions, DCs are stimulated by microbes, products of damaged tissues, cells of the innate or adaptive immune system, and inflammatory cytokines. These endogenous and exogenous antigens are taken up by DCs through the specialized endocytic system, which is mediated by a variety of receptors such as Toll-like receptors (TLRs) [4, 26], nucleotide-binding, and oligomerization domain proteins (NODs) [27, 28]. Then DCs undergo a complex process of activation making immature antigen-capturing DCs change into APCs. The process is characterized by extended dendrites of DC's external form. As a result, their cellular motility to migrate to the draining lymph node is increased [29]. Meanwhile, their surface costimulatory molecules such as CD40, CD80, and CD86 are upregulated [30–32], and MHC molecules are expressed on the surface of cells [33]. One of the major functions of DCs in immune system is capturing and presenting the antigen to T cells via MHC molecules. When the antigen is presented by APCs through MHC I, which interacts with CD8^+^ T cells, the activated T cells could differentiate into cytotoxic T lymphocytes (CTLs). Activation of CD4^+^ T cells occurs with the help of MHC II. After activation, the CD4^+^ T cells differentiate into T-helper 1 (Th1) and T-helper 2 (Th2) cells, which are involved in inducing macrophages and B-cells responses [34, 35]. CTLs are the major killers of tumor cells; they accomplish the killing with the help of CD4^+^ T cells, which can induce potential long-term CD8^+^ T-cell responses by producing various cytokines [36, 37]. 

As it is well known that intracellular endogenous antigens and exogenous antigens are presented in MHC I and MHC II by DCs, respectively [38], the strategy for DC-based vaccines in cancer immunotherapy is to make DCs cross-presentation. This can present MHC I to CTLs using internalized antigens generated from exogenous sources. Through this process, TAAs can be presented by the DC to both CD4^+^ and CD8^+^ T cells (in MHC I), and a broad and strong immune response against tumor could be induced [16, 39].

## 3. Methods Used in Genetically Engineered DCs

### 3.1. Modified/Pulsed Methods

It is generally believed that, the direct presentation of TAAs to CD8^+^ CTLs (e.g., direct administration of tumor peptides) is most tolerogenic [40, 41]. Several evidences showed that the differentiation and maturation of DCs were suppressed by cytokines existing in the tumor microenvironment. Since DCs play a crucial role in inducing antigen-specific T-cell responses, it is important to deliver tumor antigens with CD8^+^ and CD4^+^ T-cell epitopes to DCs. There are several strategies to induce DCs to present exogenous antigens on MHC I molecules [42]. 

According to the mechanism of MHC-mediated antigen presentation, early researchers tried to pulse DCs directly with tumor-specific peptides. Synthetic MHC I-binding peptides have been used in DC-based vaccination. The TAAs, which were derived from MHC I-binding peptides, including melanoma-related antigens [43, 44], carcinoembryonic antigen (CEA) [45, 46], folate binding protein (FBP) [47, 48], prostate-specific membrane antigen (PMSA) [49, 50], and Mucin 1 (MUC-1) [27, 51, 52], were firstly used to modify DCs. This strategy was easy to perform and was shown to be successful in some animal studies and clinical trials. Further development in using Tat peptide, which is from transduction domain of Human Immunodeficiency Virus (HIV), makes this strategy more effective [46, 53]. However, as this approach was mainly based on specific MHC I-restricted peptides, the importance of MHC II-restricted T-helper (Th) cells in mediating response (CD4^+^ T) and accelerating immune responses [54] was not fully considered.

Because of the limitations of using one single TAA peptide to modify DCs, researchers began to design vaccination utilizing DCs pulsed with whole tumor lysates. In this approach, as the tumor cell preparations contain lots of relevant antigens, broad-spectrum TAAs including unknown ones were presented to T cells and this induced an immune response. The advantages of this method include (1) presenting multiple peptides and epitopes to T cells to induce CTL response; (2) generating both CD8^+^ response and CD4^+^ helper T-cell response, which help to induce macrophages and B-cell response as well as prolonging the CTLs response; (3) reducing the workload of discovering and preparing appropriate peptides and epitopes required to be presented on DCs and then to be identified by T cells; (4) probably generating tumor lysate-specific memory T cells [55]. The results in animal models and in clinic trials using total tumor lysate approaches have been demonstrated to be highly effective and have low toxicity in a variety of cancers [56, 57]. It was further observed that DCs pulsed with apoptotic tumor cell preparation showed a more pronounced effect in activating T cells [58]. The main limitation of this method is the limited amount of patient tumor cells we could collect, making the preparation work difficult. Another problem is that the presence of many irrelevant antigens in tumor cell preparations could cause autoimmune responses.

Since the early reports on transfection with RNA showed strong immune response against tumor [59, 60], mRNA-pulsed DCs have become a research hotspot these days. Because RNA-based vaccines have many advantages including easily preparation, low price, specificity, control, and no risk of incorporation into the host genome [61]. DCs were transfected with tumor mRNA encoding TAA or epitopes by using carriers [61, 62] or electroporation [63, 64]. This can also induce tumor-specific immunity *in vitro*. By transfection with RNA, DCs can express specific or total antigen on their surface and finally present them to T cells [65]. In addition, using mRNA could be a promising therapy for the patients who have only few available tumor cells for mRNA preparation, because mRNA could be produced in large amounts through noninvasive biopsy procedures [66]. Recent studies indicated that mRNA has been used not only as a source of antigen, but also as a way to stimulate DC to produce immunostimulatory molecules [67]. The main limitations of using mRNA, however are difficulty in manipulation, having lower transfer efficiency and shorter lifespan (degradated by RNases rapidly) [68].

### 3.2. Modified/Pulsed Methods

To enhance DCs antitumor efficiency, the delivery of DNA encoding TAAs, immunostimulatory molecules, cytokines, chemokines, and other stimuli has been developed in the recent years. Compared with tumor antigen loading strategies described previously, genetical engineering of DCs has some special advantages, which include: (1) bypassing the work of understanding the complex intracellular process of MHC-mediated presentation; (2) achieving the purpose of cross presentation to induce a robust immune response; (3) showing a long-term antigen expression; (4) significantly reducing the autoimmune response; (5) easier preparation; (6) stability in transduction process. However, there are also some limitations in DNA-based DC vaccination. Immunotherapy using DCs transferred by viral vectors may induce an autoimmune response and mutation. Nonviral transfer shows very low transfer efficiency. DNA strategy also has its intrinsic problems such as persisting expression and genome incorporation risk. These problems are needed to be considered and solved before it becomes a better application.

**Figure 1:**
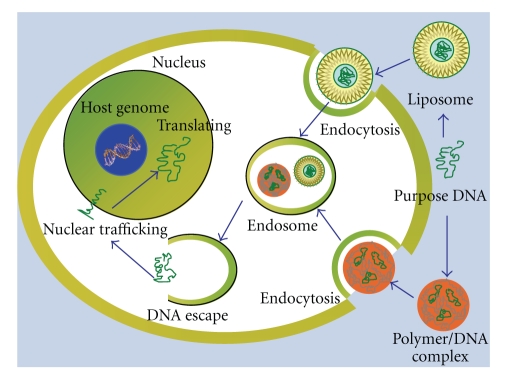
The mechanism of transfection using nonviral carriers.

**Figure 2:**
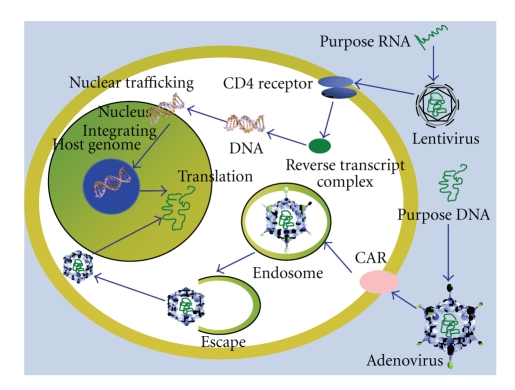
The mechanism of transfection using viral vectors.

To date, various vehicles and methods have been developed for gene transfer of DCs. Vehicles can be divided into viral vectors and nonviral carriers. And there are also some other transfection methods such as ultrasound and electroporation. By using different carries and transfection methods, the transduction efficiency and the preclinic trial results are different [69, 70]. After long-term experiments *in vitro* and *in vivo*, an increasing number of scholars think that the transduction of DCs using vehicles shows more advantages than using naked DNA alone. The major advantage of using viral vectors is their high efficiency in the transfection, which induced high protein expression levels. The limitations of this method are immune and mutation risk. For example, the host cells might express the viral proteins, and thereby might induce immunologic interference. In contrast, the nonviral strategy, which is regarded as a safer alternative to virus-mediated transduction, is considered to be promising treatment in clinic. Nonviral carriers could overcome the problems caused by viral vectors. In addition, nonviral carriers were more stable and controllable in preparation and application. The risk of virus-associated recombination mutation in host genome could be avoided. It makes DNA vaccines feasible in clinical application. But the major limitations of nonviral carriers are low efficiency in transfection and low levels of protein expression. It needs to be optimized by the carriers modified system. The mechanisms of transfection using viral vectors and nonviral carriers are shown below (Figures 1 and 2). 

#### 3.2.1. DCs Transferred by Viral Vectors

In contrast with direct viral vaccine, DCs transferred by viral vectors *in vitro* would reduce the production of certain type of antibodies, which may cause side effects and would finally reduce the effectiveness of cancer therapy in clinic [71]. The viruses employed as gene vectors include adenoviruses (Ad), adeno-associated viruses (AAV), retrovirus, lentivirus, and other viruses. These viruses are all deleted critical genes needed for their reproduction. And then, they are inserted with purpose genes such as genes encoding TAAs. Recombinant viral vectors might be the most attractive gene transduction vehicles because of their high transfection efficiency, although they are more immunogenic than nonviral carriers in clinical applications [72]. To minimize the risk of specific immunity and to boost the clinical antitumor response, several improvements in viral strategies have been developed, such as replacing the genes required for viral replication with the helper plasmids and modifying the genome of viral capsid [73]. However, to date, there is not a perfect treatment for tumor by using viral vectors-transferred DCs, as the application of viruses *in vivo* could destroy the antigen-presenting function of DCs.

**Adenovirus**

Adenovirus (Ad), which is nonenveloped and medium-sized (90–100 nm) viruses, has a double-stranded linear DNA genome. The adenoviral genome comprises four early (E1, E2, E3, and E4), four intermediate, and one late transcriptional units. To use Ad as a gene-transferring vector, the E1 gene, which contributes to reproduction, must be deleted. Ad vectors (AdVs) are based on substitution of the E1 region by the therapeutic gene. AdVs are widely used for basic and clinical research because of their high transduction efficiency [74–76]. The primary and secondary binding receptors of AdVs, Coxsackie adenovirus receptor (CAR), and V-integrin play important roles in mediating the uptake of immature DCs. AdVs can consistently induce potent presentation of both MHC class I and class II-restricted epitopes. Several studies demonstrated that using AdVs could be regarded as a valuable gene delivery system even in clinical application, for example, serotype 5 (rAd5) [77–80]. Interactions between AdVs and DCs were also investigated recently. These include virus-mediated DC maturation, antigen processing machinery (APM) regulation and T-cell activation. It was observed that the phenotype and cytokine profile of DC transduced with Ads changed [81, 82], some selected modification of DCs by Ads are listed in Table 1. These results provide the evidence for the designing human cancer vaccines.

**Retrovirus and Lentivirus**

Retrovirus is a single-stranded (ss) RNA virus, which is replicated from RNA to DNA by the revertase. Then the produced DNA is integrated into the host's genome by an integrase enzyme. With the replication of host genome, the viruses reproduce as part of the host's DNA. Retroviruses also attract the DC researchers these years for their transduction capacity of bone marrow-derived DCs (BMDCs) and cord blood-derived DCs to keep their differentiation [93, 94]. The advantages of retroviral vectors used as transduction vehicles include the stable expression of full-length proteins and less immunologic responses against viral antigens because their structural proteins are not expressed [68]. However, most retroviruses are difficult to transfect nondividing cells such as mature DCs. This disadvantage limits the application of retroviruses in clinic.

Lentiviruses derived from HIVs belong to retroviruses family. It is easier for the lentivirus to infect nondividing cells compared to other retroviruses because of its unique route of viral transfection by expressing both integrase [95] and Vpx proteins which interact with components of the nuclear pore complex [96, 97]. Another advantage of lentivirus is the low prevalence of HIV infections, which lead relatively rare pre-existing immune conditions [98]. Thus, recombined Lentivirus vectors (LVs) could be designed as efficient vectors for transduction of both mature and immature DCs. Recent studies have demonstrated that, in comparison with lipofection, electroporation and AAV, LV is the most effective vector for transduction of BMDCs [99]. Many studies reported that the highly efficient transduction of DCs *in vivo* [100–102] and *ex vivo* [103] is possible by using LVs, this can be also used for shRNA transduction [104, 105]. Moreover, LVs can also be used to transfect monocytes before differentiating into DCs, which could bypass the preactivation agents such as granulocyte-macrophage colony-stimulating actor (GM-CSF) and interleukin (IL)-4 [106]. Further modified work has been performed these years and may be extended to clinical trials. However, gene therapy using LVs has a possible risk of insertional mutagenesis.

#### 3.2.2. DCs Transferred by Nonviral Carriers

Although viral vectors have been demonstrated to be more efficient in gene delivery, the clinical application is limited due to their risk in safety and unexpected adverse effects [107]. In contrast, nonviral vectors, such as various liposomes and polyion complexes, have been increasingly developed these years because of their low immune response and ease of synthesis under controllable conditions and ease to be modified. The major limitations are their inefficient transfer, low gene expression and relatively high cytotoxicity by nature. Here is the advanced development in several nonviral vectors.

**Liposomes**

Liposomes are artificial closed vesicles of lipid bilayer membranes. Liposomes, modified with specific targeting molecular structures on surface, can be used as transfection vectors for DCs. After the APCs interact with the targeting liposomes which contain antigen peptides or DNA, the APC-mediated CTL responses are effectively enhanced [108]. Different formulations of liposomes are designed to improve the uptake by DCs through different receptor-mediated routes. These formulations of liposomes include liposomes prepared with mannosylated phosphatidylethanolamine (Man-PE), trimethyl ammonium propane [2], and phosphatidylserine [109] corresponding to mannose receptor (MR), negatively charged surface proteins and PS receptor of DCs, respectively [110].

Over the last decade, several studies have demonstrated that MR-mediated gene transfer into macrophages and DCs using mannosylated cationic liposomes can elicit effective immune responses [111–113]. MR is a typical receptor of C-type lectins, which are structurally related to surface-bound nonspecific pattern recognition receptors on the surface of monocytes, macrophages, and DCs. Using the affinity of MR with mannose-containing ligands, researchers prepared several mannosylated cationic liposomes to encapsulate DNA or RNA for gene delivery purpose. It was also reported that the transfection efficiency of macrophages and DCs was enhanced by a combination method using mannosylated lipoplexes and bubble liposomes (BLs) with ultrasound exposure. In the liver and spleen, the transfection efficiency by using this combination method was higher than that of naked pDNA or combination of unmodified lipoplexes and BLs [114]. Moreover, besides DNA delivery, siRNA silencing of DCs with liposomes is also widely used [115, 116].

Fusogenic liposome (FL) encapsulating DNA is a novel biological strategy to deliver antigen gene directly into the cytoplasm of DCs via membrane fusion. It has been demonstrated that FL-mediated OVA-gene delivery can induce potent presentation of antigen via the MHC class I-dependent pathway *in vitro* and then can induce a series of immune responses [117]. Complexes of lipoplexes with pH-sensitive fusogenic liposomes can not only transfect various malignant cells, but also can transfect a murine DC line (DC2.4) [118]. These complexes exhibited higher transfection efficiency to DC2.4 cells than some other commercially available reagents. So these new complexes may be valuable for the transfection of DCs.

**Complex Particles**

Recently, complex particles such as cationic polymers have been used as promising vectors for DNA delivery, because of their electrostatic interactions and ease of modification in targeting ligands. The advantages of cationic polymers used as gene carriers include (1) compression of the DNA into complex particles with small size and high density, which makes gene easier to transfer into cells; (2) electrostatic attraction with the cell membrane to facilitate endocytosis; (3) stability under the electrostatic repulsion. Nowadays, chitosan and biodegradable microparticles such as poly (ethylene-imine) (PEI), and so forth, attracted considerable attention in this field.

Chitosan has been used as gene delivery carrier because of good biocompatibility and high positive charge density in recent years [119]. The transfection efficiency of chitosan depends on its molecular weight, DNA complexes charge ratio, pH, and particle sizes as well as the type of cells [120, 121]. To overcome the weakness of chitosan such as poor solubility, low rate of DNA release and low efficiency of transfection, hydrophilic, and hydrophobic structure modification have been carried out. Although unmodified chitosan may not be a good gene delivery carrier for DCs because of its low transfection efficiency, some modified chitosan showed better behavior in delivering genes into DCs. For example, to enhance the IL-12 gene delivery to DCs *in vivo*, mannosylated chitosan (MC), which is used to induce mannose receptor-mediated endocytosis, was prepared to encapsulate IL-12 gene into DCs. MC not only has good physicochemical properties and low cytotoxicity, but also shows much more enhanced transfection efficiency to DCs rather than unmodified chitosan *in vitro*. And tumor growth in mouse model was suppressed by intratumoral injection of MC/plasmid encoding murine IL-12 [122, 123]. Moreover, Zhou et al. [124] developed MC microspheres containing PEI/DNA complexes, and used this carrier to improve the delivery of DNA into DCs. After *in vivo* immunization, the microspheres induced significantly enhanced serum antibody and cytotoxic T-lymphocyte (CTL) responses. Therefore, MC-mediated cytokine gene delivery system on DCs may be a potential approach for cancer immunotherapy.

Biodegradable microparticles which are easily cleared by physiological clearance systems can avoid the possible cytotoxicity caused by accumulation in cells and tissues. Microparticles prepared from poly (lactide) [125], poly(lactide-coglycolide) (PLGA), poly (orthoesters) (POE), and other polymers microparticles have been well studied in recent years. Their biodegradability, biocompatibility, and low toxicity properties make them suitable carriers for DNA vaccines. The virus-associated risk of adverse effects can also be avoided because these microparticles do not incorporate into the host cell's nucleus. Several studies have demonstrated that immune responses are induced by particle-DNA vaccines. Recently, it was reported that PLGA/PEI-DNA complex nanospheres have been developed as an efficient delivery system for the DCs. And the efficiency can be significantly promoted by modifying with nuclear localization signal (NLS) [126]. Also, such material as POE with lower cytoxicity was used to encapsulate plasmids and it induced both cellular and humoral responses *in vivo* [127]. The internalization of the particles into DCs is through phagocytosis, and the microparticles are easily phagocytosed by DC *in vitro* or* in vivo* [128].

It is well known that PEI is the most effective nonviral carrier for gene delivery. It has relative high transfection capacity due to its characteristics such as its ease in combining with DNA, binding with the cell and escaping from the endosome. Nowadays, the hotspots of research gradually focused on reducing its toxicity by various modifications. It is mainly because, cytotoxicity increases as the molecular weight increases, while the efficiency of gene loading and transfection increases correspondingly. Recently, Ali and Mooney [129] demonstrated that it showed sustained and long-term presentation when DCs were transfected with the PEI condensed with gene encoding GM-CSF. And they also use polymer PLG as scaffold fabrication continuously stimulated DCs with both GM-CSF and PEI-DNA. This process led to a 20-fold increase in gene expression than no scaffold groups, and 10 days expression *in vitro*. These results largely encouraged the development of biomaterials, such as PEI, coordinated with other macroporous scaffolds as a transfer system for DC-based vaccination. Besides, PEI-based nanoparticles could also be used to encapsulate siRNA to transfer DCs against tumor cells [130].

**Transfection Systems for Nonviral Carriers Used in DCs Transfer**

Although nonviral gene delivery system has many advantages as reported, it still cannot reach the high transfection efficiency as viral vectors do, and the period of transfection is also far from satisfactory. To improve the efficiency, one of the methods is optimizing the whole transfection system. For this purpose, the reverse and three-dimensional (3D) transfection systems have been proposed.

Some earlier studies demonstrated that reverse transfection method was more effective in enhancing the level and duration of gene expression than that of the conventional method on some cell lines [133, 134]. This may be due to the fact that DNA complexes can more easily transfect cells if they are in the area nearer to cells. In addition, cells tend to adhere to the surface and bottom of culture dish. Thus, attaching the DNA-complexes to the bottom of culture dishes before adding cells could enhance and prolong gene expression. Moreover, continuous interaction between DNA-complexes and cells would minimize the influence of serum in the transfection activity of DNA-complexes.

Several physical, chemical, and biochemical factors, can influence gene transfection efficiency. Increasing studies have reported that, when cells are cultured in 3D systems, the results of transfection in various cells such as MSCs [135, 136] and HEK293T [137] showed higher efficiency than results by using the conventional and reverse methods. And in 3D systems, cells also exhibited better morphology. The reasons may include (1) scaffolds provide larger surface area and space for the interaction between DNA-complexes and cells than that in two-dimensional (2D) systems. (2) DNA complexes can be fixed on the scaffolds and prevented from aggregation in 3D systems. (3) Signaling pathway can be influenced by 3D systems. Ali and Mooney [129] reported that nonviral vector PEI/pDNA was immobilized on a nonwoven fabric with reverse transfection method. The scaffold was treated with negative charges to facilitate the adsorption of cationic DNA/PEI complex. DCs were effectively transfected in this 3D system, and the level of gene expression was significantly higher than that of conventional transfection. It should be noted that, to date, the application of 3D or scaffold transfer system in transfection of DCs is still a new area. So the methods of cell seeding and the properties of the scaffold are still needed further exploration.

## 4. Genes Employed in DCs Transfer

 The DCs transferred with various genes can steadily and effectively express the proteins when DCs are refused *ex vivo*. After being transferred with TAA genes, DCs could express multiple antigens and epitopes. These antigens can be cross presented to MHC. DCs transferred with cytokines genes could produce large amounts of interleukin. Different genes encoding TAAs, cytokines, or chemokines are utilized to engineer DCs to increase immunogenicity. Subsequently, DCs transferred with the genes encoding TAAs can present the encoded proteins to MHC molecules and then to mediate T-cell responses. When DCs were transferred with genes encoding cytokines such as IL-7 or IL-12, the efficacy of generating T cells and immune response can be increased. When DCs were transferred with the genes encoding chemokines, the chemotaxis of DCs to T cells can be enhanced. Although this approach has not been used in clinic, it would be a potential strategy for genetic engineering technology on DCs. The genes used to transfer DCs are listed in Table 2.

## 5. Effective Cancer Immunotherapy Induced by Gene-Transferred DCs

In recent years, DC vaccines, especially DNA-based DC vaccines, have been the focus of attention in cancer immunotherapy. The main process is transferring DCs *in vitro*, and then implanting them *ex vivo*. Finally, the tumor-specific CTL response would be activated, and cancer cells would be suppressed. These adoptive immunotherapy approaches have been improved and have achieved partial success in the treatment of malignant melanoma [34], renal cell carcinoma [138], malignant lymphoma [139], and other malignant diseases [109, 140]. Most of them were phase I/II clinical trials. 

Although various tumor types were studied, melanoma, and prostate cancer are two predominant tumors treated by gene-modified DC vaccine [141]. The clinical studies for DC-based genetically modified vaccination include both viral and nonviral approaches. In viral vaccinations, recombinant AdVs were mainly used, but their application in clinic is limited by the biosafety concerns. In contrast, nonviral carriers are widely used in clinical trials because of their low toxicity. The major barrier of nonviral carriers is their low transfer efficiency compared with viral vectors. In addition to the gene delivery approach, the conformation of DCs and the route of administration are also considered to be the important issues. The formulation of DCs includes not only monocytederived DCs and BMDCs, but also immature and mature ones. The administration methods mainly include intradermal, intravenous, intranodal, and intratumoral delivery.

The above information suggests that DC-based vaccination against cancer is a promising approach with low adverse effects, but advanced efficacy studies need to be carried out. Despite this limitation, recombination DC vaccine would be considered as an encouraging tool to treat cancer. 

## 6. Conclusions and Future Perspective

DC vaccines can kill the cancer cells with little damage to normal cells by inducing and enhancing patient's own tumor-specific immune response. The function of DCs could be to optimize genetic modification by various TAAs or immune-modulatory molecules. Therefore, the application of *ex vivo *DC-based vaccination for cancer immunotherapy has many advantages because of its tumor-specific stimulation. However, for clinical applications using DC vaccines, lots of problems need to be solved, such as the low affinity between tumor epitopes and MHC, the frequency of vaccine delivery and immune procedures, and the difficulty to evaluate vaccines effect. Until now, many researchers used gene carriers and transfection systems in the recombination of DCs for effective cancer immunotherapy. With the development of materials science, targeted cell biology and molecular cytology, and so forth, various strategies have been introduced to optimize both viral and nonviral vectors for gene delivery into DCs. In addition, there is a requirement for further investigation in the use of the reverse and 3D systems to improve the nonviral transfection efficiency. These efforts are expected to facilitate future clinical applications of gene-modified DCs for cancer therapy.

## Figures and Tables

**Table 1:** Overview of recent studies using Ad vectors for gene transfer in DCs.

Cancer	Transfer molecule	*In vitro*	*In vivo* in animal model	Reference
Prostate	tPSMA and 4-1BBL	High IFN-production	Strong antitumor immunity	[83]
	STEAP	High IFN-production	Inhibition of tumor growth, vaccination delaying the growth of pre-established tumors	[84]

Hepatoma	mTERT	High IFN-and IL-2 production	Inhibition of the tumor growth	[85]
	hTERT	Inducing strong CTL response	Inducing anti-tumour immunity	[63]
	HCC and CD40L	Increasing DCs IL-12	Inducing complete regression of established tumors and long-term immunity against tumor recurrence	[86]
	AFP and HBsAg	Inducing CTLs killing HepG2.2.15 cell lines	Inhibition of tumor growth in immunodeficiency mice	[87]

Leukemia	Survivin and GM-CSF	Much higher activity of CTL than DCs with either	No data available	[88]
	IL-12 with tumor cell lysate	No data available	Prolonged survival time	[89]

Metastatic lung cancer	IL-12- and 4-1BBL	High IFN-production and CTLs response	Greater antitumor and antimetastatic effects than either treatment alone higher migratory abilities of DCs	[90]

Lung	livin	Inducing CTLs lysing LLC	Inducing a potent protective and therapeutic antitumor immunity	[91]

Urologic cancer cells	Survivin	Inducing CTLs against various bladder, kidney, and prostate cancer cells	No data available	[92]

**Table 2:** Genes used for modification of DCs.

Groups	Genes coding factors	Effects
TAAs	Gp100, MART-1, PSA, CEA, MUC-1, p53, OVA, LAMP	Lastingly expressing tumor antigens to induce the adverse effects of T-cells special response
Cytokines	IL-2, IL-7, IL-12, IL-15, IL-18 [131, 132], IL-21, IL-23, IFN, TNF-*α*	To enhance the activity of antigen-presenting function of DCs,
Chemokines	CCL21, CCL22, XCL1, CXCL9, CXCL19, CX3CL1	To guide lymphocytes to the lymph nodes
		To have angiostatic activity
Costimulatory and adhesion molecules	CD40L, CD70, 4-1BBL, OX40L RANKL, CD54, CD58, CD80	To enhance APC's ability to generate antitumor immune responses
		To improve adhesion interaction between DCs and T cells
